# Endovascular management of acute superior mesenteric vein thrombosis: a retrospective study on thrombolysis outcomes

**DOI:** 10.1186/s42155-025-00558-7

**Published:** 2025-05-14

**Authors:** Nan Wei, René Michael Mathy, De-Hua Chang, Martin Loos, Uta Merle, Annika Gauss, Monica Boxberger, Philipp Mayer, Miriam Klauss, Hans-Ulrich Kauczor, Osman Öcal, Mark O. Wielpütz

**Affiliations:** 1 Translational Lung Research Center (TLRC), German Lung Research Center (DZL), University of Heidelberg, Im Neuenheimer Feld 130.3, Heidelberg, 69120 Germany; 2 Department of Diagnostic and Interventional Radiology, University Hospital of Heidelberg, Im Neuenheimer Feld 420, Heidelberg, 69120 Germany; 3 Department of Diagnostic and Interventional Radiology With Nuclear Medicine, Thoraxklinik at University Hospital of Heidelberg, Röntgenstraße 1, Heidelberg, 69126 Germany; 4 Institute of Radiology and Nuclear Medicine, Cantonal Hospital Lucerne, Spitalstrasse, Lucerne, CH-6000 Switzerland; 5 Department of General, Visceral and Transplantation Surgery, University Hospital of Heidelberg, Im Neuenheimer Fel 420, Heidelberg, 69120 Germany; 6 Department of Gastroenterology and Hepatology, University Hospital of Heidelberg, Im Neuenheimer Feld 410, 69120 Heidelberg, Germany; 7 Department of Radiology and Neuroradiology, Greifswald University Hospital, Ferdinand-Sauerbruch-Strasse 1, Greifswald, 17475 Germany

**Keywords:** Acute Mesenteric Ischemia, Acute Superior Mesenteric Vein Thrombosis, Endovascular Treatment, Thrombolysis

## Abstract

**Background:**

Acute superior mesenteric vein thrombosis (ASMVT) is a rare but life-threatening condition associated with high morbidity and mortality. While anticoagulation remains the standard treatment, endovascular therapies such as thrombolysis, thrombectomy, and angioplasty are increasingly utilized in selected cases. However, evidence on their outcomes remains limited. This study retrospectively reports the clinical outcomes of ASMVT patients treated with endovascular combination therapies.

**Methods:**

Between August 2019 and May 2024, 12 patients (males = 9; mean age, 52.33 ± 12.51 years) were diagnosed with ASMVT. The study collected comprehensive data on demographic details, presenting symptoms, etiology, treatment modalities, response to treatment, and follow-up outcomes. Computed Tomography (CT) was available from diagnosis, and an average of 6.3 CT scans with a median follow-up of 3 months (IQR: 2–20 months).

**Results:**

The average time from symptom onset to angiographic treatment initiation was 8.0 ± 4.71 days, preceded by anticoagulation with heparin from the time of diagnosis. Patients were treated with combination therapy involving endovascular thrombolysis, rheolytic thrombectomy, and balloon angioplasty via transjugular (*n* = 9, 75%) or percutaneous (*n* = 3, 25%) approaches. Thrombolysis was performed with an average recombinant tissue plasminogen activator (rt-PA) infusion duration of 2.75 ± 1.14 days and a total dose of 61.25 ± 18.48 mg rt-PA. Superior mesenteric vein (SMV) flow was initially restored almost completely in 58.3% and partially in 41.7% of patients. Complications observed were hepatic artery bleeding (*n* = 2, 16.7%), hepatic arteriovenous fistula (*n* = 1, 8.3%), hepatic parenchymal bleeding (*n* = 1, 8.3%), melena (*n* = 1, 8.3%), and nostril bleeding (*n* = 1, 8.3%). Two patients experienced worsening symptoms of post-intervention, leading to bowel resection revealing intestinal necrosis. SMV patency was almost complete in 25%, and partially in 25% of patients at follow-up.

**Conclusion:**

Endovascular combination therapy with long-term thrombolysis and thrombectomy in patients with ASMVT demonstrated promising technical outcomes. In view of complications, individual indication for intervention needs to be confirmed in a multidisciplinary team.

## Background

Mesenteric Vein Thrombosis (MVT) is defined as acute thrombosis of the superior mesenteric vein (SMV) and its branches, with or without thrombus extension to the portal vein. It is the least common form of acute mesenteric ischemia (AMI), accounting for about one-sixth of AMI cases [1–4]. Typically, it affects the SMV and rarely impacts the inferior mesenteric vein [5].

Acute superior mesenteric vein thrombosis (ASMVT) typically presents with clinical symptoms within 24–72 h [6]. Initial signs can include classic "pain out of proportion to examination," primarily centered in the mid-abdomen, and if left untreated, ASMVT can lead to significant adverse clinical outcomes, such as bowel necrosis and death, with a reported mortality rate of up to 50% [7].

Currently, anticoagulant therapy is the standard treatment for ASMVT, primarily aimed at halting thrombus progression and preventing recurrence. According to the *Management of the Diseases of Mesenteric Arteries and Veins: Clinical Practice Guidelines of the European Society of Vascular Surgery* (ESVS), *ACG Clinical Guideline: Disorders of the Hepatic and Mesenteric Circulation and*, and Baveno VII consensus, anticoagulation is recommended as the first-line initial treatment approach [8–10]. However, for patients with extensive thrombotic burden, progressive symptoms, or those unresponsive to anticoagulation therapy, the efficacy of anticoagulation alone remains suboptimal. The management of ASMVT continues to pose a significant clinical challenge, as the overall mortality rate with traditional anticoagulation and bowel resection remains alarmingly high at 13–50% [11]. Therefore, it is particularly crucial to explore more effective alternative treatment strategies for these patients.

In light of these challenges, according to the ESVS recommendations for the treatment of MVT, endovascular treatment may be considered for patients who experience persistent symptoms, worsening abdominal pain after initiating anticoagulation, or signs of peritonitis, especially if they are poor candidates for surgery [8]. Over the past few years, minimally invasive interventional procedures (such as catheter-directed thrombolysis, mechanical thrombectomy, and balloon angioplasty) have been proposed as treatment options for ASMVT by percutaneous transhepatic (PT) and transjugular intrahepatic (TI) routes [12–14]. These techniques can directly remove or dissolve thrombus, rapidly restore mesenteric blood flow, and significantly reduce the damage caused by intestinal ischemia.

Nonetheless, large-scale evidence regarding the efficacy and safety of interventional treatment for ASMVT remains limited. Specifically, studies comparing different interventional approaches and combined treatment strategies are sparse, and standardized protocols have yet to be established. Our study aims to report the clinical outcomes of 12 patients with ASMVT who were treated at our institution through transjugular, percutaneous trans-hepatic, or trans-splenic approaches with a combination of local thrombolysis, rheolytic thrombectomy, and percutaneous transluminal angioplasty (PTA).

## Materials and methods

### Patients group

At our institution, the indication for endovascular therapy (a combination of catheter-directed thrombolysis / rheolytic thrombectomy / ballon angioplasty) via a TIPSS access or direct puncture of the portal vein is considered in the presence of risk factors (cirrhosis in transplant candidates, extensive thrombosis, known coagulopathy, portal vein stenosis, contraindication to anticoagulation) or in cases of persistent or worsening symptoms and thrombus progression despite anticoagulation as observed in a follow-up computed tomography (CT) scan.

Between August 2019 and May 2024, we treated 12 patients with ASMVT through either the transjugular (*n* = 9, 75%) or percutaneous (*n* = 3, 25%) pathway (females = 3, 25%, males = 9, 75%) with a mean age of 52.33 ± 12.51 years (range 33–70 years). For every patient, we collected the following information: demographic data, presenting symptoms, date of symptom onset, hospitalization and diagnosis, potential causes of the thrombosis, treatment and response to treatment, duration of hospitalization, laboratory test results, and outcomes at the last available follow-up.

### Radiologic evaluation

**Fig. 1:**
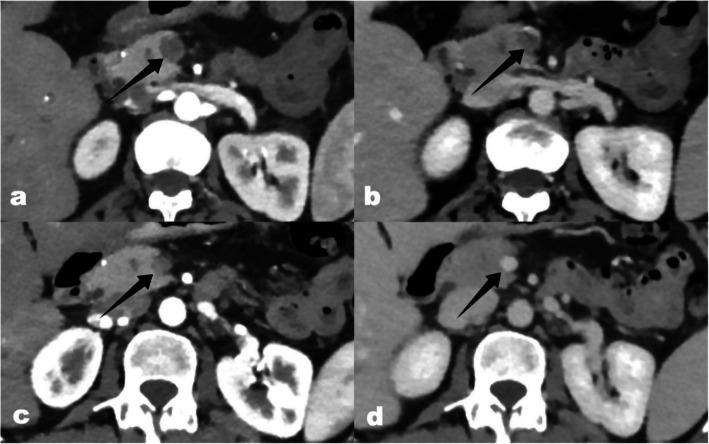
Preoperative enhanced-CT (**a**) arterial phase and (**b**) venous phase SMV thrombosis is visible (black arrow); 2 months follow-up enhanced-CT (**c**) arterial phase and (**d**) venous phase complete removal SMV thrombus (black arrow); SMV superior mesenteric vein

All 12 patients underwent contrast-enhanced CT scans within 3 days before interventional treatment and were diagnosed with ASMVT. Figure 1 shows contrast-enhanced CT images from a representative patient. The time from onset of initial symptoms to diagnosis was 2 days (range 1–4 days). Four patients were diagnosed at a local hospital, while the remaining eight were diagnosed at our institute.

### Endovascular therapy

**Fig. 2:**
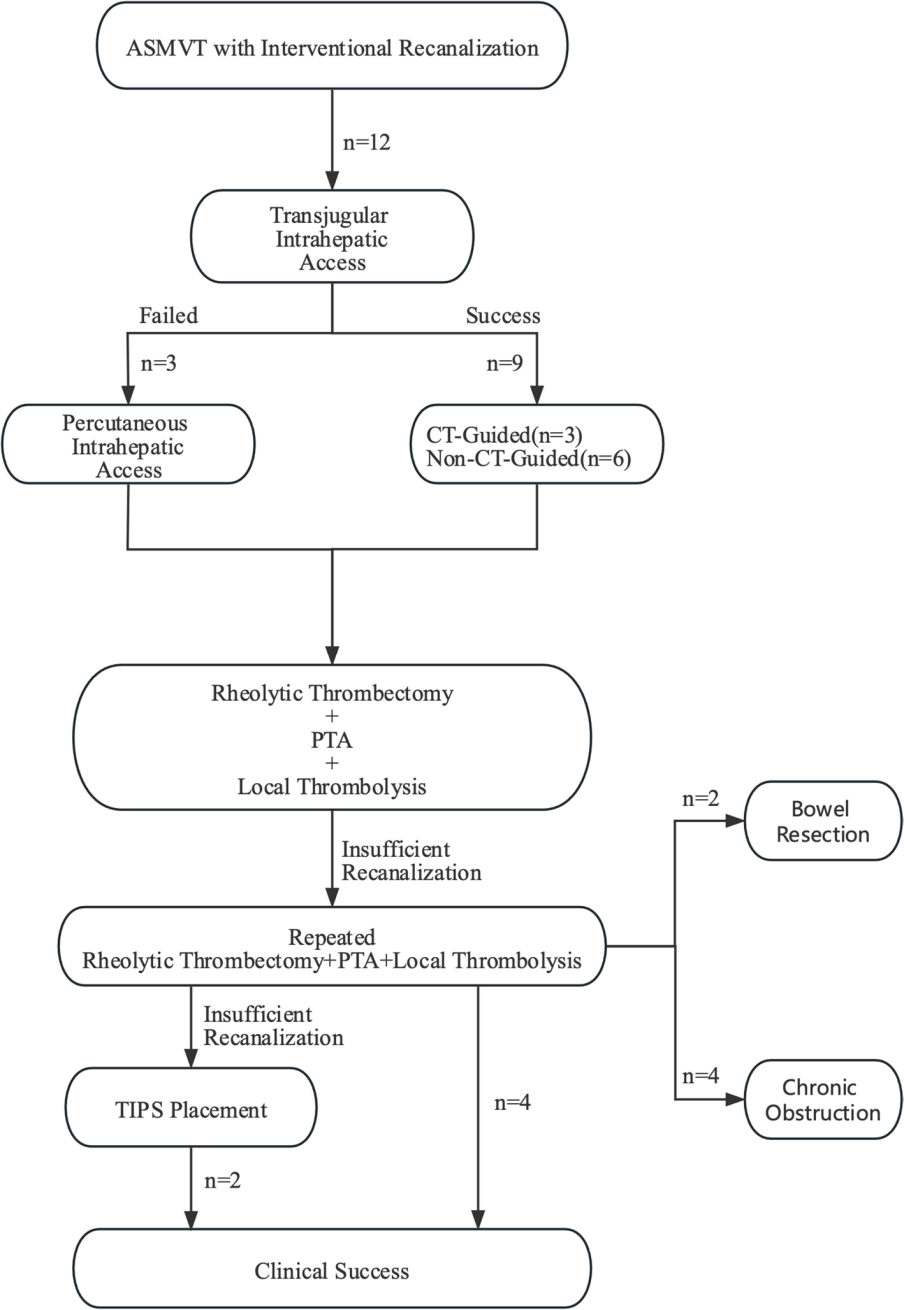
Algorithm of interventional therapy in patients with ASMVT. ASMVT acute superior mesenteric vein thrombosis; PTA percutaneous transluminal angioplasty; TIPS transjugular intrahepatic portosystemic shunt

An overview of the procedural workflow is presented in Fig. 2. All patients initially underwent a standardized treatment regimen, including fasting, nasogastric suction, intravenous hydration, broad-spectrum prophylactic antibiotics (e.g., Tazobactam and Ampicillin / Sulbactam), and intravenous heparin administration. Heparin dosages were adjusted to maintain the activated partial thromboplastin time (APTT) ratio between 2.0 and 2.5 times the control value. Contraindications to percutaneous thrombolysis included prior stroke, primary or metastatic central nervous system malignancies, active bleeding diathesis, recent gastrointestinal bleeding, evidence of intestinal ischemia or perforation, anatomic obstacles (e.g., complete portal vein thrombosis (PVT), portal cavernoma, or significant liver atrophy), and high risk of bleeding. Informed consent was obtained from every patient at least 24 h before MVT catheter-directed thrombolysis.

**Fig. 3:**
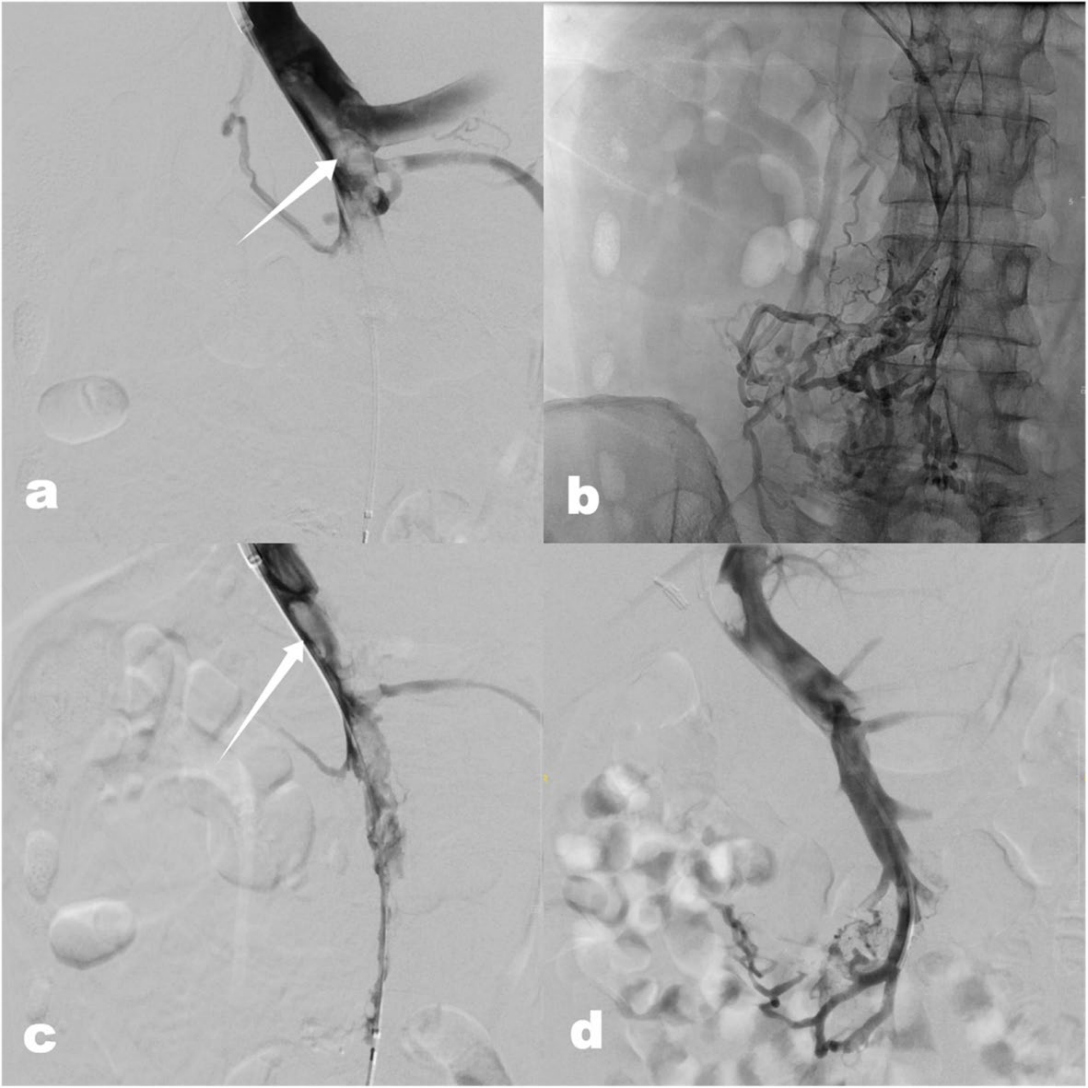
**a** Before PTA: extensive PVT (white arrow) and complete occlusion of the SMV; **b** After PTA: partial visualization of the SMV and its branches; **c** Before rheolytic thrombectomy: large amounts of thrombosis still visible in the main portal vein and SMV (white arrow); **d** After treatment for two days and before the removal of the thrombolysis catheter: a significant reduction in PVT. PTA percutaneous transluminal angioplasty; PVT portal vein thrombosis; SMV superior mesenteric vein;

For the transjugular approach illustrated in Fig. 3, patients were placed under general anesthesia and positioned on the DSA table (Artis Zee, Siemens Healthineers, Erlangen, Germany) with continuous vital sign monitoring. After sterile preparation and local anesthesia, a 10F short sheath (Terumo, Japan) was introduced over a J-wire, followed by a 5F Multipurpose or Cobra catheter (COOK Medical, USA), which was placed in the right hepatic vein. Subsequently, a 10F Checkflo sheath (COOK Medical, USA) was advanced over a superstiff wire (Boston Scientific, USA). The portal vein was punctured using a TIPSS hepatic access set (COOK Medical, USA or Optimed, Germany). After successful portal vein access, a hydrophilic wire (Terumo, Japan) was advanced into the SMV. The Multipurpose catheter was exchanged over a 0.035″ superstiff wire (Boston Scientific, USA), and an 8/60 MARS balloon catheter (Boston Scientific, USA) was used for PTA. The sheath was advanced into the portal vein towards the confluence. If a 35 cm Checkflo sheath was too short, it was replaced with a 45 cm sheath. In two patients, a TIPSS stent (Viatorr, GORE, USA) was additionally deployed.

If the transjugular approach failed, a CT-guided percutaneous method was employed. In this approach, the abdominal liver puncture area was aseptically prepared and anesthetized with 10 ml of lidocaine. A 21G Chiba needle (COOK Medical, USA) was used to puncture a portal vein branch, followed by insertion of a 4F PTCD sheath (Neff Percutaneous Access Set, COOK Medical, USA) over a micro-guidewire. Successful portal vein puncture was confirmed fluoroscopically. This method was used in three cases to assist transjugular access or as the primary interventional access in another three patients.

After vascular access was established, 5000 IU of heparin was flushed into the portal venous system. A 5F, 120 cm multipurpose catheter was advanced through the thrombus into the distal SMV until collaterals were reached. A direct thrombolysis with 10 mg rt-PA was performed during catheter retraction. Rheolytic thrombectomy (AngioJet, Boston Scientific) was then performed using a 6F, 120 cm catheter (Solent Omni, Boston Scientific) over a 0.035-inch superstiff guidewire. The device was advanced and retracted through the SMV, its branches, and the splenic vein, if necessary, until a total thrombectomy volume of 250 ml was achieved. PTA was performed if blood flow restoration in the SMV was deemed inadequate, using MARS balloons (Boston Scientific) of varying diameters (6–10 mm), as measured based on the SMV diameter. A 4F, multi-side-hole infusion catheter (Uni-Fuse, Angiodynamics, Queensbury, NY, USA) with a treatment length of 10–30 cm was placed into the thrombosed SMV for catheter-directed thrombolysis and secured with the sheath sutured in place. The Checkflo sheath was left in place during thrombolysis to allow for potential re-intervention and to maintain vascular access for heparin infusion, while thrombolytic agents were administered via the infusion catheter. If required, two infusion catheters were deployed in different SMV branches or the splenic vein.

Thrombolytic therapy was administered through the catheter at a rate of 1 mg rt-PA per hour, with fibrinogen levels monitored every 4 h. If fibrinogen levels fell below 2 g/L, the dosage was reduced to 0.5 mg rt-PA per hour. SMV angiography was performed every 24 h through the infusion catheter to evaluate treatment progress. Contrast-enhanced CT imaging was conducted 2 days post-procedure, before catheter removal, and at discharge. Catheter-directed thrombolysis was discontinued when patients exhibited significant symptomatic improvement (e.g., resolution of abdominal pain, distension, and anorexia), were able to transition to oral anticoagulation, and/or follow-up angiography and CT confirmed complete or near-complete resolution of SMV thrombosis. During hospitalization, intravenous unfractionated heparin therapy was continued to maintain the APTT ratio between 2.0 and 2.5 times the control value, transitioning to long-term rivaroxaban upon discharge.

### Study endpoints and definitions

The endpoints of this study were focused on assessing clinical outcomes and complications. Technical success was delineated as the effective deployment of an SMV catheter, achieving at least a 50% reduction in thrombus burden, and restoration of blood flow through the SMV. Clinical success was identified by symptom resolution and the obviation of the need for postoperative intestinal resection. Short-term indicators of success included a 30-day patient survival rate, amelioration of abdominal pain, and the requirement for exploratory surgery. Evaluation of long-term outcomes was based on survival rates and the recurrence of abdominal pain or SMV patency rate during follow-up outpatient visits or hospital admissions. Minor complications were classified as temporary, self-resolving symptoms with no enduring clinical consequences, whereas major complications necessitated additional interventions, hospitalization, or led to permanent adverse outcomes. Ascites was classified according to the guidelines from the International Ascites Club as mild, moderate, or severe based on imaging and clinical criteria [15]. PVT was categorized according to the Yerdel classification [16].

## Results

### Patient characteristics

The symptoms are listed in Table 1, with abdominal pain being the most common symptom experienced by all 12 patients. All patients were hemodynamically stable. Identifiable etiological factors were found in 10 (83.3%) patients, as listed in Table 1. None of the patients had underlying cirrhosis.

**Table 1:** Summary of clinical data

Patient Number	Age/Sex	Route	Symptoms	Etiologies	Indication for intervention	Days from symptoms to intervention	Complication	Length of hospital stay (days)	Length of follow-up (months)
1	52/M	TI	Moderate abdominal pain, Nausea, Anorexia, Belching, Constipation	Post-appendectomy	Constipation, continued acuity of symptoms, despite anticoagulation,	7	Right nostril bleeding	6	NA
2	47/F	TI	Moderate abdominal pain, Anorexia, Nausea	Protein S deficiency	Peritonitis, worsening pain, despite anticoagulation	5	Hepatic artery bleeding	13	2
3	38/M	TI	Moderate abdominal pain, Nausea, Distension, progressively worsened	Unknow	Peritonitis, progressive pain, despite anticoagulation	2	None	22	NA
4	60/F	TI	Severe abdominal pain, Postprandial pain, Distension, Anorexia	*JAK2-V617F* mutation DVT history	Persistent pain, despite anticoagulation	7	Hepatic artery bleeding	25	2
5	33/M	PT	Severe abdominal pain, Fever, Nausea, Vomiting, Diarrhea, Jaundice, Rigors, Distension	Antithrombin III deficiency Cerebral Venous Sinus Thrombosis History	Peritonitis, continued pain, despite anticoagulation	6	Hepatic arteriovenous fistula	21	4
6	51/F	TI	Severe abdominal pain, Nausea, Distension, progressively worsened	Thrombophilia	Worsening pain, despite anticoagulation	15	None	35	1
7	35/M	TI	Moderate abdominal pain, Nausea, Distension, Anorexia	*Factor V* mutation	Intestinal obstruction, worsening pain, despite anticoagulation	7	Melena	15	3
8	64/M	TI	Severe abdominal pain, Fever, Rigors, Constipation, Jaundice, Diarrhea, Vomiting	*JAK2-V617F* mutation *ASXL1/ZRSR2* mutation MPNs	Constipation, continued acuity of symptoms, despite anticoagulation	18	None	19	37
9	68/M	PT	Severe abdominal pain, Fever, Distension, Anorexia	Post-Whipple Surgery	Continued pain, despite anticoagulation	2	None	38	20
10	45/M	PS	Moderate abdominal pain, Postprandial pain, Nausea, Vomiting, progressively worsened	Lupus anticoagulant: *Positive*	Persistent pain, despite anticoagulation	12	None	21	50
11	70/M	TI	Moderate abdominal pain, Nausea, Vomiting, Constipation, progressively worsened	*JAK2 V617F* mutation *Factor V* mutation	Worsening pain, despite anticoagulation	10	None	60	2
12	65/M	TI	Moderate postprandial pain, Distension, Fever, progressively worsened	Unknow	Continued pain, despite anticoagulation	5	Hepatic vein bleeding	15	NA

*ASXL1* Additional sex combs like 1, *MPN M*yeloproliferative neoplasms, *NA* Not available, *PS* Percutaneous trans-splenic, *PT* Percutaneous trans-hepatic, *TI* Transjugular intrahepatic, *ZRSR2* Zinc finger RNA-binding motif and serine/arginine rich 2

Among these patients, five (41.7%) presented with ascites at initial presentation, including one (8.3%) with severe ascites, one (8.3%) with moderate ascites, and three (25%) with mild ascites, highlighting the severity of the SMV thrombosis condition. Among the 12 patients, 10 (83.3%) had concurrent PVT, which was classified as complete in 7 patients and partial in the remaining 3 patients. Additionally, PVT involved both intra- and extrahepatic regions in 3 patients, while it was confined to the extrahepatic region in the other 7 patients. The average duration from symptom onset to catheter-directed thrombolysis treatment was 8.0 ± 4.71 days, and the average follow-up time after discharge was 3 months (IQR: 2–20 months), during which patients underwent an average of 6.3 CT scans.

### Technical success

In this study, 12 patients successfully underwent thrombus aspiration of the SMV using the rheolytic thrombectomy system, combined catheter-directed continuous infusion thrombolysis with rt-PA and expansion PTA balloon dilation, thereby restoring the main flow of the SMV. Following this procedure, a catheter was left in place in the SMV to continue thrombolysis treatment for a duration of 1 to 5 days (average of 2.75 ± 1.14 days), with an average total dose of rt-PA infusion being 61.25 ± 18.48 mg (ranging from 40–80 mg). The duration of the rt-PA infusion is detailed in Table 2. After the completion of the rt-PA, six (50%) patients showed significant improvement, achieving at least a 50% reduction in the filling of the SMV, as confirmed by repeated angiography. However, two (16.7%) patients experienced an increase in thrombus post-intervention, leading to worsening symptoms and eventually, intestinal necrosis, necessitating exploratory laparotomy and bowel resection. Additionally, ascites detected in five (41.7%) patients before treatment had resolved by the time of discharge. Regarding additional treatments, two patients underwent TIPS stent placement.

**Table 2:** Patient with ASMVT infusion of rt-PA and laboratory clinical outcome

Patient number	Thrombectomy	Thrombolytic agent	Lytic Agent Dose (mg)	Duration of UK infusion (days)	Outcome	Clinical results	Surgery	Ascites	INR	APTT (s)	CRP (mg/dl)	Lactate (mg/dl)	p-Amylase (U/L)	D-Dimer (µg/L)	Before PVT Classification	After PVT Classification
1	Rheolytic thrombectomy + PTA	rt-PA	50	2	SMV thrombus continues to obstruction	Unsuccessful	None	None	1.14	24.6	161.1	8.8	NA	4.1	II	II
2	Rheolytic thrombectomy + PTA	rt-PA	55	2	Complete recanalization of the SMV	Successful	None	None	1.01	21.8	174.6	15.1	NA	NA	IV	I
3	Rheolytic thrombectomy + PTA	rt-PA	70	4	SMV thrombus continues to grow	Unsuccessful	Bowel resection	None	1.17	21.8	111.2	16.3	18	NA	III	IV
4	Rheolytic thrombectomy	rt-PA	70	3	Partial recanalization of the SMV	Successful	None	None	1.14	20.5	67.7	9	8	NA	IV	II
5	Rheolytic thrombectomy	rt-PA	30	1	SMV thrombus continues to obstruction	Unsuccessful	None	None	1.16	22.2	44.3	13.4	47	27.88	III	IV
6	Rheolytic thrombectomy + PTA	rt-PA	80	4	Significant reduction in the SMV thrombosis and partial recanalization	Successful	None	Severe	1.13	20.8	126.8	7.6	34	5.79	IV	II
7	Rheolytic thrombectomy + PTA	rt-PA	95	5	SMV thrombus continues to obstruction	Unsuccessful	None	Mild	1.13	30.4	187.7	7.4	5	14.39	I	I
8	Rheolytic thrombectomy + PTA	rt-PA	70	3	Partial recanalization of the SMV	Successful	None	None	1.15	31.2	46.9	12.2	NA	3.36	I	I
9	Rheolytic thrombectomy + PTA	rt-PA	75	3	Complete recanalization of the SMV	Successful	None	Mild	0.96	24	157.4	12.1	28	NA	III	I
10	Rheolytic thrombectomy	rt-PA	50	2	SMV thrombus continues to obstruction	Unsuccessful	None	Moderate	1.17	25.4	100.3	18.1	40	4.27	IV	I
11	Rheolytic thrombectomy + PTA	rt-PA	40	2	SMV thrombus continues to grow	Unsuccessful	Bowel resection	Mild	1.16	26.6	124.1	13.2	80	NA	II	II
12	Rheolytic thrombectomy + PTA	rt-PA	50	2	Complete recanalization of the SMV	Successful	None	None	1.15	21.2	42.2	9.5	30	NA	III	I

*APTT* Activated partial thromboplastin time, *CRP* C-reactive protein, *INR* International normalized ratio, *NA* Not available, *PTA* Percutaneous transluminal angioplasty, *PVT* Portal vein thrombosis, *rt-PA* Recombinant tissue plasminogen activator, *SMV* Superior mesenteric vein

### Complications

Regarding complications, none of the patients experienced complications at the puncture site or percutaneous liver puncture site (such as bleeding, hematoma, or leakage) during the rt-PA infusion through the catheter. During the thrombolysis treatment period, one (8.3%) patient experienced minor nostril bleeding, which was minimal and quickly ceased without interrupting the thrombolysis treatment. Among the nine patients who underwent TI approach to access the SMV, two (16.7%) suffered from hepatic artery injury leading to hemorrhage, and one (9.1%) patient developed a hepatic arteriovenous fistula. All of them were timely managed with transcatheter arterial embolization, with follow-up CT scans confirming the occlusion of vascular lesions without further complications. One (8.3%) patient developed melena during thrombolytic therapy, which resolved after discontinuing the therapy, and one (8.3%) patient suffered hepatic vein bleeding.

### Long-term outcome

After undergoing interventional treatment, 5 (41.7%) of these patients developed chronic portal vein occlusion, 4 (33.3%) had complete resolution of their PVT, and one (8.3%) patient experienced progression of the thrombosis. Clinically, all 12 patients exhibited symptom relief within 24 h of thrombectomy and thrombolysis treatment, primarily marked by a gradual reduction in abdominal pain and bloating. Among them, 12 patients achieved technical success, 6 patients achieved clinical success, and no recurrence of SMV thrombosis. The remaining 6 patients experienced continued thrombus growth, which prevented further symptom improvement and necessitated intestinal resection. Continuous clinical improvement was observed during the thrombolysis treatment via the SMV infusion catheter. The duration of hospital stay ranged from 6 to 60 days (average of 25.00 ± 14.75 days), with some patients undergoing surgical procedures for other reasons or experiencing extended hospital stays due to post-operative complications. During the follow-up period, all 12 patients were alive. All patients commenced long-term anticoagulation therapy with oral rivaroxaban for at least 6 months (ranging from 6–12 months) post-discharge, during which no repeat episodes of SMV thrombosis occurred.

## Discussion

ASMVT is a rare but potentially life-threatening condition [3]. Currently, anticoagulation remains the primary treatment for patients with ASMVT and is effective in most cases [17]. However, 5% of ASMVT patients still experience no symptom relief or even symptom worsening despite systemic anticoagulation therapy [18]. For example, in the study by Sun et al., systemic anticoagulation failed to improve conditions in up to 50% of 58 patients [19]. Therefore, this suggests that alternative or combined treatment strategies may be needed. In our study, endovascular treatment of ASMVT had 100% technical success and 50% clinical success, with only 2 (16.6%) patients requiring additional surgical measures.

In recent years, several studies have explored the application of similar invasive treatment methods in ASMVT. Although large-scale randomized controlled trials are still lacking, some small case series and retrospective studies have indicated that this combined treatment strategy may offer potential benefits for certain patients. For example, Li et al. studied 23 patients with non-cirrhotic PVT, using a combination of TI route and rheolytic thrombectomy, showing a 100% technical success rate and significant symptom improvement [20]. Another study by Yang et al. reviewed 14 patients using a stepwise treatment strategy combining thrombolysis and rheolytic thrombectomy, also achieving positive outcomes [14]. Wang et al. studied 12 patients using rheolytic thrombectomy combined with local thrombolysis via the TI route, showing rapid blood flow restoration and significantly improved prognosis [21]. Additionally, Kim et al. analyzed 11 patients and confirmed that transhepatic catheter-directed thrombectomy and thrombolysis could rapidly relieve symptoms and reduce the incidence of long-term complications [1]. The TI approach is preferred for thrombolysis as it avoids puncturing the hepatic or splenic capsules, thereby lowering the risk of subcapsular hemorrhage associated with capsule traversal and subsequent thrombolysis and anticoagulation in the percutaneous trans-hepatic or trans-splenic approach [11, 21, 22]. The TI approach should be prioritized when feasible, as it reduces the risk of direct liver and splenic injury and peritoneal puncture, which is especially beneficial for patients with ascites or coagulation disorders, also provides the option for TIPS placement if needed, which has been shown to improve patency [23].

In our study, we employed an escalating invasive combination therapeutic strategy aimed at rapidly restoring blood flow in the SMV and preventing complications such as intestinal ischemia and necrosis. This approach began with rheolytic thrombectomy, followed by local thrombolysis (including bolus and overnight lysis), and balloon angioplasty. The primary objective of PTA is to maximize the restoration of patency in the SMV and to eliminate residual luminal stenosis after rheolytic thrombectomy. This is because rheolytic thrombectomy or thrombolysis often leaves behind residual thrombus or irregularities of the intimal surface, leading to persistent luminal narrowing, resulting in sluggish blood flow and an increased risk of recurrent thrombosis. When necessary, stenting was performed, and if these methods failed to achieve adequate flow restoration, TIPS implantation was considered to improve the outflow, especially in cases with thrombus reaching the portal vein. However, we must cautiously evaluate the efficacy and safety of this treatment approach. Firstly, radiological assessments revealed that 6 patients (50%) demonstrated a reduction of more than 50% in SMV filling defects after rt-PA thrombolysis, indicating limited effectiveness of the treatment in achieving vascular recanalization. Additionally, 2 patients (16.7%) experienced exacerbation of thrombosis and symptom deterioration following the intervention, ultimately developing intestinal necrosis that required exploratory laparotomy and bowel resection. These surgeries were performed under ongoing thrombolytic therapy, which increased the risk of perioperative bleeding. This underscores a major limitation of percutaneous treatment: the inability to directly assess intestinal viability, potentially delaying the timely identification and management of intestinal necrosis. In addition, while TIPS implantation can be an effective option for restoring blood flow, it comes with certain risks, especially in patients with preexisting liver disease [24]. For those with conditions like cirrhosis, TIPS may worsen liver function by increasing the hepatic burden, potentially leading to further complications [24]. Moreover, TIPS can also aggravate right heart failure, particularly in patients with existing right heart dysfunction [24]. Therefore, careful assessment of the patient’s overall condition is necessary before deciding whether to proceed with this intervention.

Current guidelines remain cautious about the widespread application of invasive treatments [8]. This caution primarily arises from the significant risks of complications associated with such treatments. Patients with liver cirrhosis often receive anticoagulation therapy alone due to the risk of hepatic decompensation and the relatively high likelihood of spontaneous recanalization. In a prospective study conducted by Nery et al., approximately 70% of cases with partial PVT in cirrhotic patients experienced spontaneous recanalization during follow-up [25]. Therefore, treatment strategies for non-cirrhotic patients are generally more aggressive, whereas those for cirrhotic patients are more cautious and individualized. The Baveno VII guidelines provide recommendations on the management of PVT and splanchnic thrombosis in both cirrhotic and non-cirrhotic patients. In non-cirrhotic patients, immediate therapeutic anticoagulation is recommended for recent PVT or splanchnic vein thrombosis (SVT), which continued for at least 6 months. Long-term anticoagulation is advised for those with a persistent prothrombotic state and may be considered in others. For past PVT or SVT, including incomplete resolution after 6 months, long-term anticoagulation is similarly recommended. However, invasive treatments should be considered in cases with ASMVT with progressing symptoms against its risks. For example, while catheter-directed thrombolysis and mechanical thrombectomy are effective in restoring blood flow, they are linked to severe complications such as bleeding, infection, and organ failure. A meta-analysis by Rodrigues et al. indicated that the higher rates of major complications and mortality observed in studies involving thrombolysis may be attributed to factors such as the choice of access routes, a higher proportion of cavernous transformation, and more extensive thrombosis in non-cirrhotic PVT patients [26]. Yang et al. reported that 4 patients (50%) required additional surgical intervention following thrombolytic therapy due to persistent and unremitting abdominal distention, whereas in our study, only 2 patients (16.7%) underwent bowel resection, which is comparatively lower. This discrepancy may be attributed to differences in the timing of intervention, as the average interval from symptom onset to hospitalization in Yang et al.'s cohort was 12.63 ± 3.62 days, whereas in our study, the average time from symptom onset to angiographic treatment initiation was shorter at 8.0 ± 4.71 days, potentially allowing for earlier intervention and improved outcomes [11]. In a study by Rabuffi et al., one patient died from multi-organ failure following pharmaco-mechanical thrombectomy [12], and Kim et al. reported a case of death due to sepsis and multi-organ dysfunction after transhepatic catheter-directed thrombectomy [1]. Additionally, Li [20] observed hepatic subcapsular hematoma in 8.7% of 23 patients undergoing invasive treatments. Adding to these concerns, a study of 21 patients with PVT highlighted that 9 patients (42.8%) experienced hemorrhagic complications [23]. Additionally, in another study of 35 patients who underwent interventional procedures for PVT, 10 patients (28.6%) also experienced bleeding complications [27]. Therefore, such invasive interventions are generally not recommended unless patients exhibit worsening conditions under anticoagulation therapy, persistent symptoms, or signs of intestinal ischemia [28]. The decision to proceed with invasive treatment should be comprehensively evaluated by a multidisciplinary team (MDT) [8, 19].

Moreover, prompt recognition of patients who require surgical intervention is crucial for improving clinical outcomes [29]. The decision to perform an exploratory laparotomy should be carefully considered the high risk of bleeding associated with recent thrombolysis. Surgical intervention is generally indicated for patients presenting severe and diffuse peritonitis, transmural bowel infarction, or bowel perforation [30]. According to our treatment protocol, repeated CT scans are performed 48 h after thrombolysis, or earlier if clinical symptoms worsen, particularly in cases of increasing abdominal pain or distension, or the development of peritoneal signs. These radiologic assessments are crucial for evaluating bowel perfusion, identifying early signs of ischemia or infarction, and guiding further clinical management.

This study has several limitations, including a small sample size and limited long-term follow-up. Inherent selection bias is unavoidable, as only patients who failed anticoagulation therapy or presented with severe symptoms warranting more aggressive intervention were selected for endovascular treatment. Consequently, this study cannot yield statistically significant conclusions regarding the optimal approach, agent, or dosage for thrombolysis, nor can it thoroughly assess potential risk factors.

## Conclusion

Our study on invasive combination therapy strategies demonstrates certain potential benefits in patients with ASMVT, particularly in terms of rapid symptom relief, prevention of thrombosis recurrence, reduced mortality, and decreased need for intestinal resection. However, it is important to note that combination percutaneous treatments were performed only in a subset of ASMVT patients. Due to the lack of high-quality evidence, clear clinical guideline support, and the risk of complications, careful and cautious evaluation is required to identify patients who may benefit most from this approach. Given the low incidence of ASMVT, conducting large-scale randomized controlled trials may be impractical, future research should focus on multi-center studies or pooled data analyses to provide stronger evidence and guide clinical practice.

## Data Availability

The datasets used and/or analyzed during the current study are available from the corresponding author on reasonable request.

## Abbreviations

AMI: Acute mesenteric ischemia
APTT: Activated partial thromboplastin time
ASMVT: Acute superior mesenteric vein thrombosis
CRP: C-reactive protein
CT: Computed tomography
ESVS: European Society of Vascular Surgery
MDT: Multidisciplinary team
MPN: Myeloproliferative neoplasms
MVT: Mesenteric vein thrombosis
PT: Percutaneous transhepatic
PTA: Percutaneous transluminal angioplasty
PVT: Portal vein thrombosis
rt-PA: Recombinant tissue plasminogen activator
SMV: Superior mesenteric vein
SVT: Splanchnic vein thrombosis
TI: Transjugular intrahepatic
TIPS: Transjugular Intrahepatic Portosystemic Shunt
